# Online absolute calibration of fast FEL pulse energy measurements

**DOI:** 10.1107/S1600577523001133

**Published:** 2023-03-10

**Authors:** Pavle Juranić, Arturo Alarcon, Rasmus Ischebeck

**Affiliations:** a Paul Scherrer Institut, Forschungsstrasse 111, Villigen 5232, Switzerland; University of Tokyo, Japan

**Keywords:** free-electron lasers, FELs, shot-to-shot absolute flux measurements

## Abstract

One of the challenges facing modern free-electron laser (FEL) facilities is the accurate pulse-to-pulse online measurement of the absolute flux of the X-ray pulses, for use by both machine operators for optimization and users of the photon beam to better understand their data. This manuscript presents a methodology that combines existing slow-measurement methods currently used in gas detectors across the world and fast uncalibrated signals from multipliers, meant for relative flux pulse-to-pulse measurements, which create a shot-to-shot absolute flux measurement through the use of sensor-based conditional triggers and algorithms at SwissFEL.

## Abstract 2

A new method for online pulse-resolved absolute pulse energy measurements at free-electron lasers is presented.

## 1. Introduction

The need for an absolute online measurement of photon flux at X-ray free-electron lasers (FELs) has been apparent since the inception of these new large-scale devices. The photon pulse energy is one of the main measures of the effectiveness of the FEL setup, and is used for, among other things, gain curve measurements of the undulators, sorting of data to find non-linear effects in experiments and judging the effectiveness of different machine setups. This measurement of the pulse energy has been pioneered by the diagnostics group at the Free Electron Laser in Hamburg (FLASH) at the Deutsches Elektronen Synchrotron (DESY) and the X-ray gas monitor detector (XGMD) developed there (Sorokin *et al.*, 2019 ▸). Use of this technology as an online measurement has spread to other FELs, with similar devices now existing at facilities such as LCLS, SACLA, FERMI, European XFEL and SwissFEL (Sorokin *et al.*, 2019 ▸; Tiedtke *et al.*, 2014 ▸; Zangrando *et al.*, 2009 ▸; Grünert *et al.*, 2019 ▸; Owada *et al.*, 2018 ▸; Tono *et al.*, 2013 ▸). The accuracy of the XGMD system has been confirmed several times at various facilities with measurements against a radiative bolometer using both soft and hard X-rays (Tiedtke *et al.*, 2014 ▸; Kato *et al.*, 2012 ▸; Juranic *et al.*, 2019 ▸). The XGMD mainly measures the flux on a long time scale, evaluating the total current on a copper plate from the ions that have been photoionized and then drawn to the plate by a strong electric field. The hardware and robustness of the device ensures the accuracy of the measurement, but it delivers data on a long time scale, typically giving an average current in roughly 10 to 30 s. The XGMD has the option to measure the electron current on the plates opposite the ions and extract a shot-to-shot evaluation that can be calibrated to the pulse energy, but this feature requires a very high photon flux or a large cross-section for sufficient signal, with the latter available only for soft X-rays. The XGMD is an excellent tool to evaluate the average pulse energy, but it cannot provide a single-shot evaluation of the pulse energy for hard X-rays and low fluxes.

Another component of the gas detector system developed by DESY and used at various facilities, including SwissFEL, is the huge aperture open multiplier (HAMP), which is a large multiplier used for single-shot relative flux measurements that are not an absolute evaluation of the pulse energy. The response of this device to the ions generated from the photoionization depends on the potential that they are operated under, and the energy and charge of the photoionized ions that are impacting the HAMP surface. Furthermore, this response changes with time, as the multiplier coating slowly depletes over years of use. It is theoretically possible to evaluate the absolute single-shot pulse energy from the HAMP measurements if one can characterize the multiplier for every gas type and pressure, photon energy and voltage setting, year after year. Furthermore, the multiplier itself must be set with a voltage that has the signal generated by the ion impact to be in the linear regime. A constant monitoring of the signal amplitude must be implemented that feeds back on the multiplier voltage to ensure the operation of this device in a reliable manner. It was developed to deal with hard X-rays and lower fluxes which are encountered at most hard X-ray FEL facilities.

This manuscript describes the developments in hardware characterization, feedback and monitoring programs, and processing algorithms that allow the photon pulse energy monitor (PBIG) at SwissFEL to deliver absolute pulse energy evaluations on a shot-to-shot basis (Juranić *et al.*, 2018 ▸). The PBIG is the renamed DESY-developed and constructed pulse energy monitor, and the methods proposed here can be adapted to any similar device at FELs around the world.

## 2. Measurement setup

### 2.1. Detector reliability

The precursor to effective data processing and evaluation of pulse-resolved pulse energy is the reliability of the input data for this evaluation. The XGMD slow absolute energy measurement must be calibrated against another device, and the fast HAMP measurement has to be operating so it can react linearly to the incoming pulse energies, and hence the data collected for eventual algorithmic processing are not dominated by noise or empty measurements.

The XMGD average pulse energy measurements are linear and were calibrated in previous work (Juranic *et al.*, 2019 ▸). The copper plate from which the current is measured by a Keithley 6514 calibrated multimeter has a quantum efficiency of 1, and the multimeter has a linear measurement range for current measurements that spans more than ten orders of magnitude. This device provides the calibrated long-scale average signal that will be used to evaluate the shot-to-shot pulse energy from the HAMPs.

**Figure 1:**
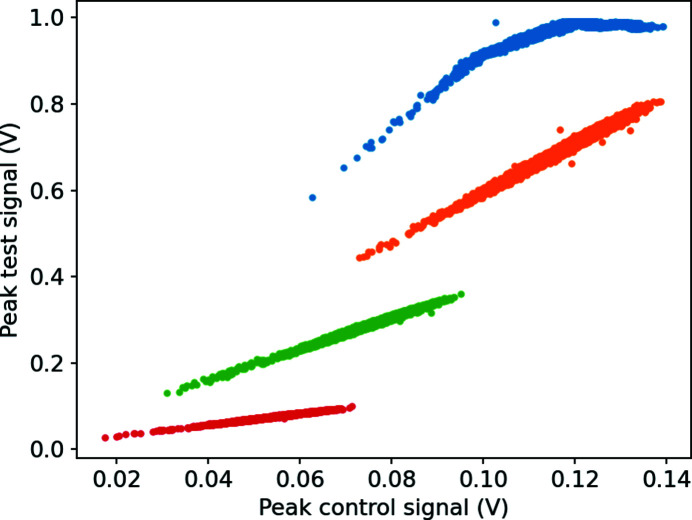
Linearity measurement of the HAMP detectors on the ADC.

The HAMPs, in contrast, need characterization to evaluate their range of linearity under an applied gain voltage. This voltage needs to be regulated through an overwatch program so that the HAMP detector signals remain linear, while also being high enough to provide a good signal-to-noise ratio on its analog-to-digital converter (ADC). An example of the ion signal on the ADC from the HAMP is presented by Sorokin *et al.* (2019 ▸). Since the response of the HAMP multiplier also changes with the photon energy, pulse energy and gas type, the most appropriate metric to observe in order to ensure linearity is the signal from the HAMP itself, or its maximum absolute peak height. The commissioning of the HAMP at SwissFEL used the fact that we have two such devices, one oriented along the vertical axis and another along the horizontal axis, and kept the settings of the horizontal (HAMP-X) constant and in the linear range, and changed the gain voltage on the vertical (HAMP-Y) to observe which peak heights are in the linear range. Further consultations with the team at DESY who built the devices concluded that the detector is linear between the maximum peak voltage of 1 mV and 10 mV, which translates to 10 mV and 100 mV on the ADC due to a 20 dB pre-amplifier between the HAMP and the 16-bit Ioxos ADC card used at the Aramis branch of SwissFEL. As shown in Fig. 1 ▸, the linear response also extends beyond this range and only begins to be non-linear once the peak value of the signal reaches around 0.9 V. The ADC maximum input voltage restricts the maximum signal strength to 1 V, resulting in the flat line once this value is reached.

The controls system at SwissFEL reads the shot-to-shot voltage peaks from the HAMPs, and if the peak reading is above 100 mV or below 10 mV for longer than 10 s it changes the HAMP gain voltage by 50 V appropriately. This active feedback on the HAMP readings, combined with a large linear response ‘buffer’, ensures there is sufficient time during the HAMP voltage changes to ensure the device is always linearly responding to the X-ray beam pulse energy.

Further measures to ensure the reliability of the gas detector setup include a system that stops its data-processing whenever the electron and photon beams are shut off, either accidentally or intentionally. The two elements monitored to determine whether the PBIG data are processed are the bunch charge determined by the monopole cavity of an electron online beam position monitor (DBPM) in the linear accelerator, and the photon shutter. If the DBPM reading is in a valid state and can detect the electron beam, and the shutter is open, acquisition and processing can take place. If either of these two criteria is not met, the data processing is suspended, and the pulse energy reading for both the fast and the slow signals is automatically set to zero.

### 2.2. Algorithm for data-processing

The core of the data processing and evaluation of the absolute pulse energy on a shot-to-shot basis is the evaluation of the ratio between the slow signals and the fast signals. The slow absolute evaluation from the XGMD has an integration time of about 10 s, updated every second as the Keithley multimeter updates its readout. The fast signal reads out the relative pulse energy from the integral of the ion peaks at the repetition rate of SwissFEL, up to 100 Hz. To be able to compare these two evaluations with each other directly on a pulse-by-pulse basis, we first create a rolling buffer of pulse-resolved measurements that is as long as or longer than the XGMD evaluation integration time. The rolling buffer always maintains the same number of elements, adding a new element with each new processed FEL pulse, while dropping the oldest element in the buffer. The rolling buffer is updated at the repetition rate of the FEL, and is used to continuously evaluate the conversion constant *C_i_* so that

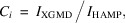

where *I*_XGMD_ and *I*_HAMP_ are the evaluations of the XGMD and HAMP signal data in the buffer, respectively. This constant is then used in further evaluations. A weighted average algorithm is used to evaluate the current conversion constant so that

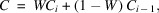

where *W* is the weighting factor, equal to the period of the FEL divided by the chosen buffer length time constant, and *C*_*i*–1_ is the previous conversion constant. A 10 s time constant and 100 Hz repetition rate would yield a weighting factor of 0.001. The role of this weighting factor and the data buffer is to ensure that the conversion constant between the XGMD and HAMP readouts is not affected by single-shot losses of pulse energies and remains stable unless the relationship between the two devices is altered due to a change in photon energy or multiplier voltage gain. The FEL radiation can vary significantly on a shot-to-shot basis owing to the stochastic nature of the self-amplified spontaneous emission (SASE), so such a large buffer is necessary to establish a suitable conversion constant between the two devices. The last step of the data processing is to evaluate the single pulse energy, which is equal to *C* × *I*_HAMP_.

If the repetition rate of the FEL changes, the rolling buffer size is recalculated to accommodate the larger number of points in the chosen time period, and the buffer itself is reset. However, the constant *C* remains unchanged unless the photon energy or the HAMP gain voltage change.

**Figure 2:**
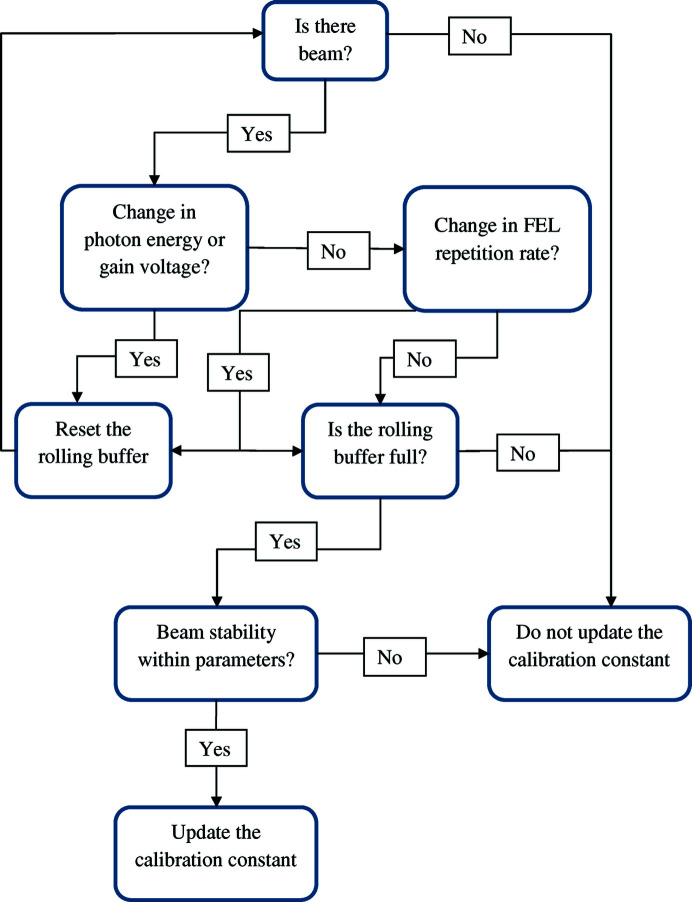
Flowchart of the data processing to produce the calibration constant *C* on a pulse-resolved basis.

The data buffer and single-shot pulse energy evaluation process is restarted when the FEL changes its photon energy or the HAMP gain voltage changes by more than 10 V, since both of these alter the ratio between the XGMD and HAMP readings. Once the rolling buffer is full, an algorithm checks the data within the rolling buffer and checks whether the data are within the stability criteria set to evaluate the ratio. In the case of SwissFEL, these stability criteria are based on the HAMP and XGMD data, with the most commonly used stability criteria being that the XGMD readings should have a peak-to-peak variance of less than 5% of the average pulse energy over the length of the rolling buffer. These criteria ensure that the conversion constant between the XGMD and HAMP readings is taken when the beam is in a stable mode, and gives an accurate evaluation of the conversion constant *C*. If the beam is not on, the rolling buffer is not full, or the beam stability is not within the set parameters, *C* is not updated, and the constant that existed up to that point is used. As long as the HAMP voltage or the photon energy does not change, *C* is constant for the calculations, as the HAMP response relative to the XGMD signal does not change. If there is no constant, no fast absolute pulse energy is displayed until the constant can be evaluated. If the beam is on, the rolling buffer is full and the beam is within the stability criteria, the calibration constant *C* updates with every pulse according to the process described above. The flowchart in Fig. 2 ▸ illustrates the data-processing flow.

## 3. Results and discussion

**Figure 3:**
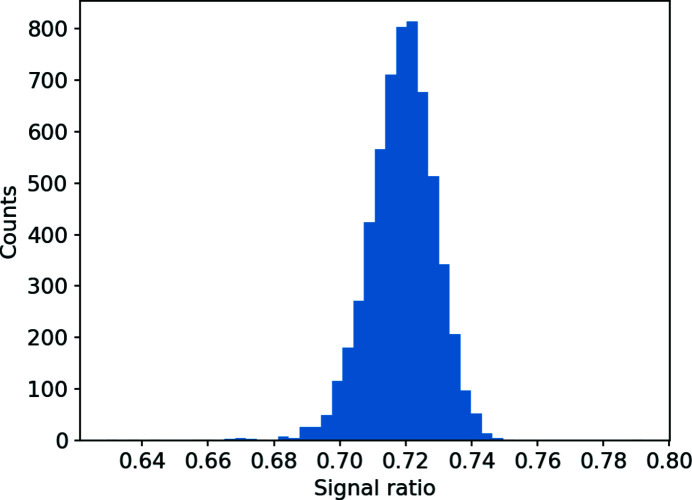
Ratio distribution of the HAMP-X versus HAMP-Y signals for 10 000 pulses during standard operation of SwissFEL.

The resulting evaluation of the absolute single-shot pulse energy matches both the absolute numbers measured by the XGMDs, and shows the shot-to-shot fluctuations of their amplitudes, as seen in Fig. 3 ▸. The fast measurement comparison was made under conditions that kept the HAMP gain voltage constant, at a constant photon energy. The ratio between the two HAMPs comes from the different detector responses. The signal yields vary between different HAMPs due to the artisanal quality of their manufacturing and coating procedure, in this case by about 30%. The standard deviation from the mean ratio of the signals from HAMP-Y versus HAMP-X was about 1.4%, which is also the relative measurement accuracy of the single-shot measurement.

**Figure 4:**
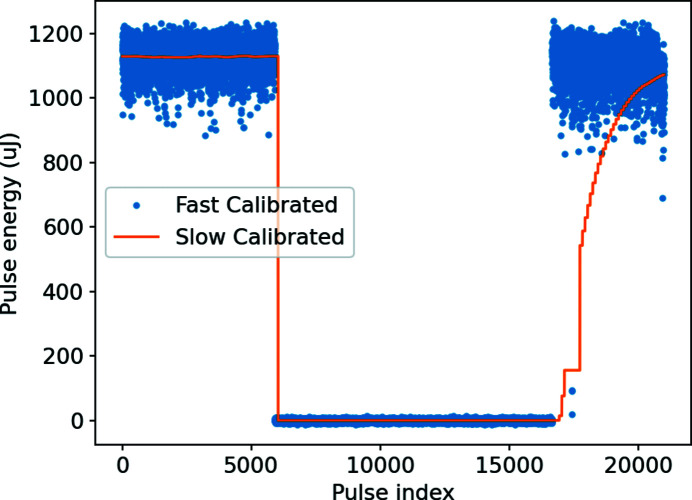
Signal recovery of the FEL lasing after an interruption due to a short-lived fault in the accelerator at a repetition rate of 100 Hz.

Additionally, the fast algorithm can react quickly to sudden drops in pulse energy, showing the sudden stop and return to lasing almost instantaneously, while the slow signal takes significant time to ramp back up, as shown in Fig. 4 ▸. This allows users who need to stop and restart their measurements to resume reliable data collection more quickly, and the operators can immediately see how well the FEL is lasing after some kind of a temporary failure, without waiting for up to 30 s. The fast signal can also be employed for faster acquisitions of gain curve scans of the undulators (Milton *et al.*, 2001 ▸) and quicker optimization algorithms for machine performance owing to a faster monitoring parameter as the main input (Kirschner *et al.*, 2022 ▸).

The method and algorithm described here have been shown to work at SwissFEL with its repetition rate of up to 100 Hz. The optimization of the algorithm to process the data has been shown to be 100% reliable even at the maximum 100 Hz repetition rate, has no skipped points and matches perfectly with other beam-synchronous measurements. Other facilities with larger repetition rates may have more difficulty in finding the time necessary between the pulses to execute full evaluations to provide a real-time single-shot pulse energy measurement. However, the algorithm can also be used to assign pulse energies to data after the fact, though some of the features such as fast online optimization and quick gain curve acquisition would be lost.

## 4. Conclusions

The development of the absolute fast pulse energy measurement is a step forward in creating a system that can be more responsive to lasing efficiency and fluctuations. Most gas-based pulse energy detectors currently offer a choice between a fast uncalibrated signal or a slow calibrated signal to investigate and optimize machine performance, both of which have downsides. A slow calibrated signal leads to a slow correction response, whereas a fast uncalibrated signal only works while the pulse energy or photon energy are within parameters that enable full functionality of the fast signals, like the HAMPs. The absolute fast pulse energy measurement ensures a fast response to both large and small changes, and would be significantly faster than the slow calibrated signal.

Though the setup described is fast, an even better setup would be one where the evaluation of the pulse energy would depend completely on values measured from the HAMPs, their gain voltage and a photon energy. This is theoretically possible, but would require a long-term project to gather sufficient data to correlate these parameters to the absolutely measured pulse energy on a shot-to-shot basis, and a setup that ensures every data point measured is valid. The scheme described in this manuscript creates such a system.

The data gathered by the fast pulse energy measurement are currently evaluated using a comparison against the slow pulse energy measurement. However, with enough time and data points, one could use this data to create a machine-learning algorithm that would enable the evaluation of the pulse energy directly, without having to compare the HAMP values with the slow calibrated XGMD signals. In that respect, the effort described here is the first step to eventually create a wholly calibrated fast pulse energy measurement for all possible beam parameters.
